# Bayesian Inference for Nonnegative Matrix Factorisation Models

**DOI:** 10.1155/2009/785152

**Published:** 2009-05-27

**Authors:** Ali Taylan Cemgil

**Affiliations:** Department of Computer Engineering, Boğaziçi University, 34342 Bebek, Istanbul, Turkey

## Abstract

We describe nonnegative matrix factorisation (NMF) with a Kullback-Leibler (KL) error measure in a statistical framework, with a hierarchical generative model consisting of an observation and a prior component. Omitting the prior leads to the standard KL-NMF algorithms as special cases, where maximum likelihood parameter estimation is carried out via the Expectation-Maximisation (EM) algorithm. Starting from this view, we develop full Bayesian inference via variational Bayes or Monte Carlo. Our construction retains conjugacy and enables us to develop more powerful models while retaining attractive features of standard NMF such as monotonic convergence and easy implementation. We illustrate our approach on model order selection and image reconstruction.

## 1. Introduction

In machine learning, nonnegative matrix factorisation (NMF) was introduced by Lee and Seung [1] as an alternative to k-means clustering and principal component analysis (PCA) for data analysis and compression (also see [2]). In NMF, given a *W* × *K* nonnegative matrix *X* = {*x*_*ν*,*τ*_}, where *ν* = 1 : *W*, *τ* = 1 : *K*, we seek positive matrices *T* and *V* such that

(1) $$x_{\nu ,\tau }\approx {\left[TV\right]}_{\nu ,\tau }=\sum_{i}t_{\nu ,i}v_{i,\tau },$$

where *i* = 1 : *I*. We will refer to the *W* × *I* matrix *T* as the *template matrix*, and *I* × *K* matrix *V* the *excitation matrix*. The key property of NMF is that *T* and *V* are constrained to be positive matrices. This is in contrast with PCA, where there are no positivity constraints or k-means clustering where each column of *V* is constrained to be a unit vector. Subject to the positivity constraints, we seek a solution to the following minimisation problem:

(2) $${\left(T,V\right)}^{*}=\text{arg}\;\underset{T,V>0}{\text{min}\;}\;D(X∥TV).$$

Here, the function *D* is a suitably chosen error function. One particular choice for *D*, on which we will focus here, is the information (Kullback-Leibler) divergence, which we write as

(3) $$D(X∥\Lambda )=-\underset{\nu ,\tau }{\sum }\left(x_{\nu ,\tau }\;\;\text{log}\frac{\lambda_{\nu ,\tau }}{x_{\nu ,\tau }}-\lambda_{\nu ,\tau }+x_{\nu ,\tau }\right).$$

Using Jensen's inequality [3] and concavity of log *x*, it can be shown that *D*(·) is nonnegative and *D*(*X*||Λ) = 0 if and only if *X* = Λ. The objective in (2) could be minimised by any suitable optimisation algorithm. Lee and Seung [1] have proposed a very efficient variational bound minimisation algorithm that has attractive convergence properties and which has been successfully applied in various applications in signal analysis and source separation, for example, [4–6].

The interpretation of NMF, like singular value decomposition (SVD), as a low rank matrix approximation is sufficient for the derivation of a useful inference algorithm; yet this view arguably does not provide the complete picture about assumptions underlying the statistical properties of *X*. Therefore, we describe NMF from a statistical perspective as a hierarchical model. In our framework, the original nonnegative multiplicative update equations of NMF appear as an expectation-maximisation (EM) algorithm for maximum likelihood estimation of a conditionally Poisson model via *data augmentation*. Starting from this view, we develop Bayesian extensions that facilitate more powerful modelling and allow more sophisticated inference, such as Bayesian model selection. Inference in the resulting models can be carried out easily using variational (structured mean field) or Markov Chain Monte Carlo (Gibbs sampler). The resulting algorithms outperform existing NMF strategies and open up the way for a full Bayesian treatment for model selection via computation of the marginal likelihoods (the evidence), such as estimating the dimensions of the template matrix or regularising overcomplete representations via automatic relevance determination.

## 2. The Statistical Perspective

The interpretation of NMF as a low-rank matrix approximation is sufficient for the derivation of an inference algorithm; yet this view arguably does not provide the complete picture. In this section, we describe NMF from a statistical perspective. This view will pave the way for developing extensions that facilitate more realistic and flexible modelling as well as more sophisticated inference, such as Bayesian model selection.

Our first step is the derivation of the information divergence error measure from a maximum likelihood principle. We consider the following hierarchical model:

(4) $$\begin{array}{l} T∼p(T\;|\;\Theta^{t}),\;\;V∼p(V\;|\;\Theta^{v}), \end{array}$$

(5) $$\begin{array}{c} s_{\nu ,i,\tau }∼𝒫𝒪(s_{\nu ,i,\tau };t_{\nu ,i}v_{i,\tau }),\;\;x_{\nu ,\tau }=\underset{i}{\sum }s_{\nu ,i,\tau }. \end{array}$$

Here, *𝒫𝒪*(*s*; *λ*) denotes the Poisson distribution of the random variable *s* ∈ *ℕ*_0_ with nonnegative intensity parameter *λ*, where

(6) 𝒫𝒪(s;λ)=exp(s  log  λ−λ−log Γ(s+1))

and Γ(*s* + 1) = *s*! is the gamma function. The priors *p*(*T* ∣ ·) and *p*(*V* ∣ ·) will be specified later. We call the variables *S*_*i*_ = {*s*_*ν*,*i*,*τ*_}* latent sources*. We can analytically marginalise out the latent sources *S* = {*S*_1_ ⋯ *S*_*I*_} to obtain the marginal likelihood

(7) $$\begin{array}{ll} \text{log}\;p(X\;|\;T,V) & =\text{log}\;\underset{S}{\sum }p(X\;|\;S)p(S\;|\;T,V)\\ & =\text{log}\;\underset{\nu ,\tau }{\prod }𝒫𝒪(x_{\nu ,\tau };\underset{i}{\sum }t_{\nu ,i},v_{i,\tau })\\ & =\underset{\nu }{\sum }\;\underset{\tau }{\sum }\left(x_{\nu ,\tau }\;\;\text{log}{\left[TV\right]}_{\nu ,\tau }-{\left[TV\right]}_{\nu ,\tau }-\text{log}\;\Gamma (x_{\nu ,\tau }+1)\right). \end{array}$$

This result follows from the well-known *superposition property* of Poisson random variables [7], namely, when *s*_*i*_ ∼ *𝒫𝒪*(*s*_*i*_; *λ*_*i*_) and *x* = *s*_1_ + *s*_2_ + ⋯ + *s*_*I*_, then the marginal probability is given by *p*(*x*) = *𝒫𝒪*(*x*; ∑_*i*_ *λ*_*i*_). The maximisation of this objective in *T* and *V* is equivalent to the minimisation of the information divergence in (3). In the derivation of original NMF in [8], this objective is stated first; the *S* variables are introduced implicitly later during the optimisation on *T* and *V*. In the sequel, we show that this algorithm is actually equivalent to EM, ignoring the priors *p*(*T* ∣ ·) and *p*(*V* ∣ ·).

### 2.1. Maximum Likelihood and the EM Algorithm

The log-likelihood of the observed data *X* can be written as

(8) $$\begin{array}{ll} {ℒ}_{X}(T,V) & \equiv \text{log}\;\underset{S}{\sum }\;p(X\;|\;S)p(S\;|\;T,V)\\ & \geq \underset{S}{\sum }q(S)\;\text{log}\frac{p(X,S\;|\;T,V)}{q(S)}\equiv {ℬ}_{\text{EM}}[q], \end{array}$$

where *q*(*S*) is an instrumental distribution, that is arbitrary provided that the sum on the right exists; *q* can only vanish at a particular *S* only when *p* does so. Note that this defines a lower bound to the log-likelihood. It can be shown via functional derivatives and imposing the normalisation condition ∑_*S*_ *q*(*S*) = 1 via Lagrange multipliers that the lower bound is tight for the exact posterior of the latent sources, that is,

(9) $$\text{arg}\;\underset{q(S)}{\text{max}\;}\;{ℬ}_{\text{EM}}[q]=p(S\;|\;X,T,V).$$

Hence the log-likelihood can be maximised iteratively as follows:

(10) $$\begin{array}{c} \text{E}\;\;\text{Step}\;q{\left(S\right)}^{\left(n\right)}=p(S\;|\;X,T^{\left(n-1\right)},V^{\left(n-1\right)}),\\ \text{M}\;\;\text{Step}\;(T^{\left(n\right)},V^{\left(n\right)})=\text{arg}\;\underset{T,V}{\text{max}\;}\;{\langle \;\text{log}\;p\left(S,X\;|\;T,V\right)\rangle }_{q{\left(S\right)}^{\left(n\right)}}. \end{array}$$

Here, 〈*f*(*x*)〉_*p*(*x*)_ = ∫ *p*(*x*)*f*(*x*)*dx*, the expectation of some function *f*(*x*) with respect to *p*(*x*). In the E step, we compute the posterior distribution of *S*. This defines a lower bound on the likelihood

(11) $${ℬ}^{\left(n\right)}(T,V\;|\;T^{\left(n-1\right)},V^{\left(n-1\right)})={\langle \;\text{log}\;p\left(S,X\;|\;T,V\right)\rangle }_{q{\left(S\right)}^{\left(n\right)}}.$$

For many models in the exponential family, which includes (5), the expectation on the right depends on the sufficient statistics of *q*(*S*)^(*n*)^ and is readily available; in fact calculating *q*(*S*) should be literally taken as calculating the sufficient statistics of *q*(*S*). The lower bound is readily obtained as a function of these sufficient statistics, and maximisation in the M Step yields a fixed point equation.

#### 2.1.1. The E Step

To derive the posterior of the latent sources, we observe that

(12) $$p(S\;|\;X,T,V)=\frac{p(S,X\;|\;T,V)}{p(X\;|\;T,V)}.$$

For the model in (5), we have

(13) $$\begin{array}{ll} & \log p(S,X\;|\;T,V)\\ & \;\;=\underset{\nu }{\sum }\;\underset{\tau }{\sum }(\underset{i}{\sum }(-t_{\nu ,i}v_{i,\tau }+s_{\nu ,i,\tau }\;\;\text{log}(t_{\nu ,i}v_{i,\tau })-\text{log}\;\Gamma (s_{\nu ,i,\tau }+1))\\ & \;\;\;\;\;\;+\text{log}\;\delta (x_{\nu ,\tau }-\underset{i}{\sum }s_{\nu ,i,\tau })). \end{array}$$

It follows from (5), (12), (13), and (7)

(14) $$\begin{array}{ll} & \text{log}\;p(S\;|\;X,T,V)\\ & \;\;=\underset{\nu }{\sum }\;\underset{\tau }{\sum }(\underset{i}{\sum }\left(s_{\nu ,i,\tau }\;\;\text{log}(\frac{t_{\nu ,i}v_{i,\tau }}{\sum_{i'}t_{\nu ,i'}v_{i',\tau }})-\text{log}\;\Gamma (s_{\nu ,i,\tau }+1)\right)\\ & \;\;\;\;\;\;+\text{log}\;\Gamma (x_{\nu ,\tau }+1)+\text{log}\;\delta (x_{\nu ,\tau }-\underset{i}{\sum }s_{\nu ,i,\tau }))\\ & \;\;=\underset{\nu }{\sum }\;\underset{\tau }{\sum }\text{log}\;ℳ(s_{\nu ,1,\tau },\ldots ,s_{\nu ,I,\tau };x_{\nu ,\tau },p_{\nu ,1,\tau },\ldots ,p_{\nu ,I,\tau }), \end{array}$$

where *p*_*ν*,*i*,*τ*_ ≡ *t*_*ν*,*i*_ *v*_*i*,*τ*_/∑_*i*'_ *t*_*ν*,*i*'_ *v*_*i*',*τ*_ are the cell probabilities. Here, *ℳ* denotes a multinomial distribution defined by

(15) $$\begin{array}{ll} ℳ(s;x,p) & =\left(\begin{array}{c} x\\ s_{1}\;s_{2}\;\cdots \;s_{I} \end{array}\right)p_{1}^{s_{1}}p_{2}^{s_{2}}\cdots p_{I}^{s_{I}}\delta (x-\underset{i}{\sum }s_{i})\\ & =\delta (x-\underset{i}{\sum }s_{i})x!\overset{I}{\underset{i=1}{\prod }}\frac{p_{i}^{s_{i}}}{s_{i}!}, \end{array}$$

where **s** = {*s*_1_, *s*_2_,…, *s*_*I*_}, **p** = {*p*_1_, *p*_2_,…, *p*_*I*_}, and *p*_1_ + *p*_2_ + ⋯ + *p*_*I*_ = 1. Here, *p*_*i*_, *i* = 1 ⋯ *I* are the cell probabilities, and *x* is the index parameter where *s*_1_ + *s*_2_ + ⋯ + *s*_*I*_ = *x*. The Kronecker delta function is defined by *δ*(*x*) = 1 when *x* = 0, and *δ*(*x*) = 0 otherwise. It is a standard result that the marginal mean is

(16) $$\langle s_{i}\rangle =xp_{i},$$

that is, the expected value of each source *s*_*i*_ is a fraction of the observation, where the fraction is given by the corresponding cell probability.

#### 2.1.2. The M Step

It is indeed a good news that the posterior has an analytic form. Since now the M step can be calculated easily as follows:

(17) $$\begin{array}{ll} & {\langle \text{log}\;p(S,X\;|\;T,V)\rangle }_{p(S|X,T,V)}\\ & =\underset{\nu }{\sum }\underset{\tau }{\sum }(\underset{i}{\sum }(-t_{\nu ,i}v_{i,\tau }+\langle s_{\nu ,i,\tau }\rangle \log(t_{\nu ,i}v_{i,\tau })-\langle \text{log}\;\Gamma (s_{\nu ,i,\tau }+1)\rangle )\\ & \;\;\;\;+\langle \text{log}\;\delta (x_{\nu ,\tau }-\underset{i}{\sum }s_{\nu ,i,\tau })\rangle ). \end{array}$$

Fortunately, for maximisation with respect to *T* and *V*, the last two difficult terms are merely constant, and we need only to maximise the simpler objective

(18) $$Q(T,V)=\underset{\nu }{\sum }\underset{\tau }{\sum }\left(\underset{i}{\sum }\left(-t_{\nu ,i}v_{i,\tau }+{\langle s_{\nu ,i,\tau }\rangle }^{\left(n\right)}\;\;\text{log}(t_{\nu ,i}v_{i,\tau })\right)\right),$$

where we only need the expected value of the sources given by the previous values of the templates and excitations:

(19) $${\langle s_{\nu ,i,\tau }\rangle }^{\left(n\right)}=x_{\nu ,\tau }\frac{t_{\nu ,i}^{\left(n\right)}v_{i,\tau }^{\left(n\right)}}{\sum_{i'}t_{\nu ,i'}^{\left(n\right)}v_{i',\tau }^{\left(n\right)}}.$$

Maximisation of the objective *Q* and substituting 〈*s*_*ν*,*i*,*τ*_〉^(*n*)^ give the following fixed point equations:

(20) $$\begin{array}{ll} \frac{\partial Q}{\partial t_{\nu ,i}} & =-\underset{\tau }{\sum }v_{i,\tau }^{\left(n\right)}+\frac{\sum_{\tau }{\langle s_{\nu ,i,\tau }\rangle }^{\left(n\right)}}{t_{\nu ,i}},\\ t_{\nu ,i}^{\left(n+1\right)} & =\frac{\sum_{\tau }{\langle s_{\nu ,i,\tau }\rangle }^{\left(n\right)}}{\sum_{\tau }v_{i,\tau }^{\left(n\right)}}=t_{\nu ,i}^{\left(n\right)}\frac{\sum_{\tau }x_{\nu ,\tau }v_{i,\tau }^{\left(n\right)}/\sum_{i'}t_{\nu ,i'}^{\left(n\right)}v_{i',\tau }^{\left(n\right)}}{\sum_{\tau }v_{i,\tau }^{\left(n\right)}},\\ \frac{\partial Q}{\partial v_{i,\tau }} & =-\underset{\nu }{\sum }t_{\nu ,i}^{\left(n\right)}+\frac{\sum_{\nu }{\langle s_{\nu ,i,\tau }\rangle }^{\left(n\right)}}{v_{i,\tau }},\\ v_{i,\tau }^{\left(n+1\right)} & =\frac{\sum_{\nu }{\langle s_{\nu ,i,\tau }\rangle }^{\left(n\right)}}{\sum_{\nu }t_{\nu ,i}^{\left(n\right)}}=v_{i,\tau }^{\left(n\right)}\frac{\sum_{\nu }t_{\nu ,i}^{\left(n\right)}x_{\nu ,\tau }/\sum_{i'}t_{\nu ,i'}^{\left(n\right)}v_{i',\tau }^{\left(n\right)}}{\sum_{\nu }t_{\nu ,i}^{\left(n\right)}}. \end{array}$$

Equation (20) is identical to the multiplicative update rules of [8]. However, our derivation via data augmentation obtains the same result as an EM algorithm. It is interesting to note that in literature, NMF is often described as EM-like; here, we show that it is actually just an EM algorithm. We see that the efficiency of NMF is due to the fact that the *W* × *I* × *K* object 〈*S*〉 needs not to be explicitly calculated as we only need its marginal statistics (sums across *τ* or *ν*).

We note that our model is valid when *X* is integer valued. See [9] for a detailed discussion about consequences of this issue. Here, we assume that for nonnegative real valued $\overset{˜}{X}$, we only consider the integer part, that is, we let $\tilde{X}=X+E$, where *E* is a noise matrix with entries uniformly drawn in [0, 1). In practice, this is not an obstacle when the entries of *X* are large.

The interpretation of NMF as a maximum likelihood method in a Poisson model is mentioned in the original NMF paper [1] and discussed in more detail by [5, 10]. The equivalence of NMF and probabilistic latent sematic analysis is shown in [11]. Kameoka in [5] focuses on the optimisation and gives an equivalent description using auxiliary function maximisation. In contrast, the auxiliary variables can be viewed as model variables (the sources *s*) that are analytically integrated out [10]. A general framework is described in [12]. Prior structures are placed on conditionally Gaussian NMF models to enforce sparsity in [13]. However, all of these approaches are based on regularisation, that is, aim at calculating a maximum a posteriori estimate. In contrast, we provided in this article a full Bayesian treatment where the templates and excitations are integrated out.

### 2.2. Hierarchical Prior Structure

Given the probabilistic interpretation, it is possible to propose various hierarchical prior structures to fit the requirements of an application. Here we will describe a simple choice where we have a conjugate prior as follows:

(21) $$t_{\nu ,i}∼𝒢(t_{\nu ,i};a_{\nu ,i}^{t},\frac{b_{\nu ,i}^{t}}{a_{\nu ,i}^{t}}),\;\;v_{i,\tau }∼𝒢(v_{i,\tau };a_{i,\tau }^{v},\frac{b_{i,\tau }^{v}}{a_{i,\tau }^{v}}).$$

Here, *𝒢* denotes the density of a gamma random variable *x* ∈ ℝ_+_ with shape *a* ∈ ℝ_+_ and scale *b* ∈ ℝ_+_ defined by

(22) $$𝒢(x;a,b)=\text{exp}((a-1)\;\text{log}\;x-\frac{x}{b}-\text{log}\;\Gamma (a)-a\;\;\text{log}\;b).$$

**Figure 1:**
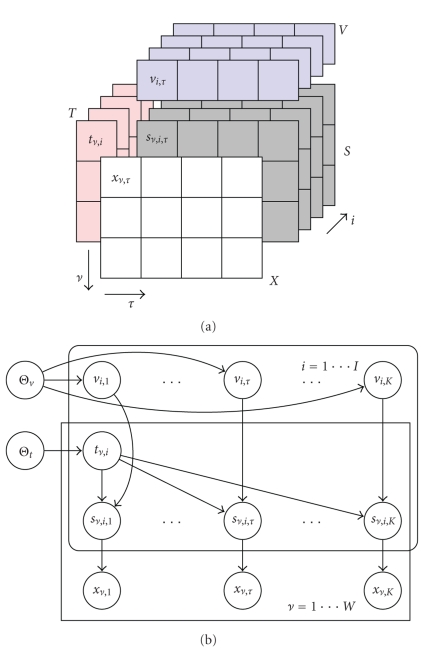
(a) A schematic description of the NMF model with data augmentation. (b) Graphical model with hyperparameters. Each source element *s*_*ν*,*i*,*τ*_ is Poisson distributed with intensity *t*_*ν*,*i*_ *v*_*i*,*τ*_. The observations are given by *x*_*ν*,*τ*_ = ∑_*i*_ *s*_*ν*,*i*,*τ*_. In matrix notation, we write *X* = ∑ *S*_*i*_. We can analytically integrate out over *S*. Due to superposition property of Poisson distribution, intensities add up, and we obtain 〈*X*〉 = *TV*. Given *X*, the NMF algorithm is shown to seek the maximum likelihood estimates of the templates *T* and excitations *V*. In our Bayesian treatment, we further assume that elements of *T* and *V* are Gamma distributed with hyperparameters Θ.

**Figure 2:**
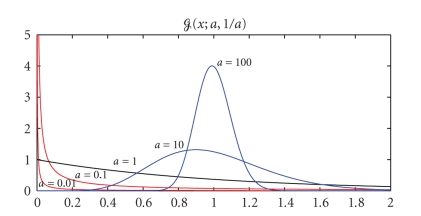
(Left) The family of densities *p*(*v*; *a*, *b* = 1) = *𝒢*(*v*; *a*, *b*/*a*) with the same mean 〈*v*〉 = *b* = 1. Small values of *a* (for *a* < 1) enforce sparser representations, and large values of *a* ≈ 100 tie all values to be close to a nonzero mean (nonsparse representation).

The primary motivation for choosing a Gamma distribution is computational convenience: Gamma distribution is the conjugate prior to Poisson intensity. The indexing highlights the most general case where there are individual parameters for each element *t*_*ν*,*i*_ and *v*_*i*,*τ*_. Typically, we do not allow many free hyperparameters but tie them depending upon the requirements of an application. See Figure 1 for an example. As an example, consider a model where we tie the hyperparameters such as *a*_*ν*,*i*_^*t*^ = *a* ^*t*^, *b*_*ν*,*i*_^*t*^ = *b* ^*t*^, *a*_*i*,*τ*_^*v*^ = *a* ^*v*^, and *b*_*i*,*τ*_^*v*^ = *b* ^*v*^ for *i* = 1 ⋯ *I*, *ν* = 1 ⋯ *W*, and *τ* = 1 ⋯ *K*. This model is simple to interpret, where each component of the templates and the excitations is drawn independently from the Gamma family shown in Figure 2. Qualitatively, the shape parameter *a* controls the *sparsity* of the representation. Remember that *𝒢*(*x*; *a*, *b*/*a*) has the mean *b* and standard deviation $b/\sqrt{a}$. Hence, for large *a*, all coefficients will have more or less the same magnitude *b*, and typical representations will be full. In contrast, for small *a*, most of the coefficients will be very close to zero, and only very few will be dominating, hence favouring a sparse representation. The scale parameter *b* is adapted to give the expected magnitude of each component.

To model missing data, that is, when some of the *x*_*ν*,*τ*_ are not observed, we define a *mask* matrix *M* = {*m*_*ν*,*τ*_}, the same size as *X* where *m*_*ν*,*τ*_ = 0, if *x*_*ν*,*τ*_ is missing and 1 otherwise (see Appendix A.4 for details). Using the mask variables, the observation model with missing data can be written as

(23) $$p(X\;|\;S)p(S\;|\;T,V)=\underset{\nu ,\tau }{\prod }{\left(p(x_{\nu ,\tau }\;|\;s_{\nu ,1:I,\tau })p(s_{\nu ,1:I,\tau }\;|\;t_{\nu ,1:I},v_{1:I,\tau })\right)}^{m_{\nu ,\tau }}.$$

The hierarchical model in (21) is more powerful than the basic model of (5), in that it allows a lot of freedom for more realistic modelling. First of all, the hyperparameters can be estimated from examples of a certain class of source to capture the invariant features. Another possibility is Bayesian model selection, where we can compare alternative models in terms of their marginal likelihood. This enables one to estimate the model order, for example, the optimum number of templates to represent a source.

## 3. Full Bayesian Inference

Below, we describe various interesting problems that can be cast to Bayesian inference problems. In signal analysis and feature extraction with NMF, we may wish to calculate the posterior distribution of templates and excitations, given data and hyperparameters Θ ≡ (Θ^*t*^, Θ^*v*^). Another important quantity is the marginal likelihood (also known as the *evidence*), where

(24) $$p(X\;|\;\Theta )=\int dT\;\;dV\underset{S}{\sum }p(X\;|\;S)p(S\;|\;T,V)p(T,V\;|\;\Theta ).$$

The marginal likelihood can be used to estimate the hyperparameters, given examples of a source class

(25) $$\Theta^{*}=\arg\underset{\Theta }{\max}\;\;p(X\;|\;\Theta )$$

or to compare two given models via *Bayes factors*

(26) $$l(\Theta_{1},\Theta_{2})=\frac{p(X\;|\;\Theta_{1})}{p(X\;|\;\Theta_{2})}.$$

This latter quantity is particularly useful for comparing different classes of models. Unfortunately, the integrations required cannot be computed in closed form. In the sequel, we will describe the Gibbs sampler and variational Bayes as approximate inference strategies.

### 3.1. Variational Bayes

We sketch here the Variational Bayes (VB) [3, 14] method to bound the marginal log-likelihood as

(27) $$\begin{array}{ll} {ℒ}_{X}(\Theta ) & \equiv \text{log}\;p(X\;|\;\Theta )\geq \underset{S}{\sum }\int d(T,V)q\;\;\text{log}\frac{p(X,S,T,V\;|\;\Theta )}{q}\\ & ={\langle \text{log}\;p\left(X,S,V,T\;|\;\Theta \right)\rangle }_{q}+H[q]\equiv {ℬ}_{\text{VB}}[q], \end{array}$$

where *q* = *q*(*S*, *T*, *V*) is an instrumental distribution, and *H*[*q*] is its entropy. The bound is tight for the exact posterior *q*(*S*, *T*, *V*) = *p*(*S*, *T*, *V* ∣ *X*, Θ) but as this distribution is complex, we assume a factorised form for the instrumental distribution by ignoring some of the couplings present in the exact posterior as follows:

(28) $$\begin{array}{ll} q(S,T,V) & =q(S)q(T)q(V)\\ & =\left(\underset{\nu ,\tau }{\prod }q(s_{\nu ,1:I,\tau })\right)\left(\underset{\nu ,i}{\prod }q(t_{\nu ,i})\right)\left(\underset{i,\tau }{\prod }q(v_{i,\tau })\right)\equiv \underset{\alpha \in 𝒞}{\prod }q_{\alpha }, \end{array}$$

where ***α*** ∈ *𝒞* = {{*S*}, {*T*}, {*V*}} denotes set of disjoint clusters. Hence, we are no longer guaranteed to attain the exact marginal likelihood *ℒ*_*X*_(Θ). Yet, the bound property is preserved, and the strategy of VB is to optimise the bound. Although the best *q* distribution respecting the factorisation is not available in closed form, it turns out that a local optimum can be attained by the following fixed point iteration:

(29) $$q_{\alpha }^{\left(n+1\right)}\propto \text{exp}\left({\langle \text{log}\;p\left(X,S,T,V\;|\;\Theta \right)\rangle }_{q_{\neg \alpha }^{\left(n\right)}}\right),$$

where *q*_*¬****α***_ = *q*/*q*_***α***_. This iteration monotonically improves the individual factors of the *q* distribution, that is, *ℬ*[*q* ^(*n*)^] ≤ *ℬ*[*q* ^(*n*+1)^] for *n* = 1, 2,… given an initialisation *q* ^(0)^. The order is not important for convergence; one could visit blocks in arbitrary order. However, in general, the attained fixed point depends upon the order of the updates as well as the starting point *q* ^(0)^(·). We choose the following update order in our derivations:

(30) $$q{\left(S\right)}^{\left(n+1\right)}\propto \text{exp}\left({\langle \text{log}\;p\left(X,S,T,V\;|\;\Theta \right)\rangle }_{q{\left(T\right)}^{\left(n\right)}q{\left(V\right)}^{\left(n\right)}}\right),$$

(31) $$q{\left(T\right)}^{\left(n+1\right)}\propto \text{exp}\left({\langle \text{log}\;p\left(X,S,T,V\;|\;\Theta \right)\rangle }_{q{\left(S\right)}^{\left(n+1\right)}q{\left(V\right)}^{\left(n\right)}}\right),$$

(32) $$q{\left(V\right)}^{\left(n+1\right)}\propto \text{exp}\left({\langle \text{log}\;p\left(X,S,T,V\;|\;\Theta \right)\rangle }_{q{\left(S\right)}^{\left(n+1\right)}q{\left(T\right)}^{\left(n+1\right)}}\right).$$

### 3.2. Variational Update Equations and Sufficient Statistics

The expectations of 〈log *p*(*X*, *S*, *T*, *V* ∣ Θ)〉 are functions of the sufficient statistics of *q* (see the expression in the Appendix A.2). The update equation for the latent sources (30) leads to the following:

(33) $$\begin{array}{ll} q(s_{\nu ,1:I,\tau }) & \propto \text{exp}(\underset{i}{\sum }(s_{\nu ,i,\tau }(\langle \text{log}\;t_{\nu ,i}\rangle +\langle \text{log}\;v_{i,\tau }\rangle )-\text{log}\;\Gamma (s_{\nu ,i,\tau }+1)))\delta (x_{\nu ,\tau }-\underset{i}{\sum }s_{\nu ,i,\tau })\\ & \propto ℳ(s_{\nu ,1,\tau },\ldots ,s_{\nu ,i,\tau },\ldots ,s_{\nu ,I,\tau };\\ & \;\;\;x_{\nu ,\tau },p_{\nu ,1,\tau },\ldots ,p_{\nu ,i,\tau },\ldots ,p_{\nu ,I,\tau }),\\ & p_{\nu ,i,\tau }=\frac{\text{exp}(\langle \text{log }t_{\nu ,i}\rangle +\langle \text{log }v_{i,\tau }\rangle )}{\sum_{i}\;\exp(\langle \text{log }t_{\nu ,i}\rangle +\langle \text{log }v_{i,\tau }\rangle )},\\ & \;\;\;\langle s_{\nu ,i,\tau }\rangle =x_{\nu ,\tau }p_{\nu ,i,\tau }. \end{array}$$

These equations are analogous to the multinomial posterior of EM given in (14); only the computation of cell probabilities is different. The excitation and template distributions and their sufficient statistics follow from the properties of the gamma distribution:

(34) $$\begin{array}{cc} q(t_{\nu ,i}) & \propto \text{exp}(\left(a_{\nu ,i}^{t}+\underset{\tau }{\sum }\langle s_{\nu ,i,\tau }\rangle -1)\log(t_{\nu ,i}\right)\\ & \;-\left(\frac{a_{\nu ,i}^{t}}{b_{\nu ,i}}+\underset{\tau }{\sum }\langle v_{i,\tau }\rangle \right)t_{\nu ,i})\\ & \propto 𝒢\left(t_{\nu ,i};\alpha_{\nu ,i}^{t},\beta_{\nu ,i}^{t}\right),\\ \alpha_{\nu ,i}^{t} & \equiv a_{\nu ,i}^{t}+\underset{\tau }{\sum }\langle s_{\nu ,i,\tau }\rangle ,\;\beta_{\nu ,i}^{t}\equiv {\left(\frac{a_{\nu ,i}^{t}}{b_{\nu ,i}^{t}}+\underset{\tau }{\sum }\langle v_{i,\tau }\rangle \right)}^{-1},\\ \exp(\langle \log\;t_{\nu ,i}\rangle ) & =\text{exp}(\Psi (\alpha_{\nu ,i}^{t}))\beta_{\nu ,i}^{t},\\ \langle t_{\nu ,i}\rangle & =\alpha_{\nu ,i}^{t}\beta_{\nu ,i}^{t},\\ q(v_{i,\tau }) & \propto \text{exp}((a_{i,\tau }^{v}+\underset{\nu }{\sum }\langle s_{\nu ,i,\tau }\rangle -1)\text{log}\;v_{i,\tau }\\ & \;\;-\left(\frac{a_{i,\tau }^{v}}{b_{i,\tau }^{v}}+\underset{\nu }{\sum }\langle t_{\nu ,i}\rangle \right)v_{i,\tau })\\ & \propto 𝒢\left(v_{i,\tau };\alpha_{i,\tau }^{v},\beta_{i,\tau }^{v}\right),\\ \alpha_{i,\tau }^{v} & \equiv a_{i,\tau }^{v}+\underset{\nu }{\sum }\langle s_{\nu ,i,\tau }\rangle ,\;\beta_{i,\tau }^{v}\equiv {\left(\frac{a_{i,\tau }^{v}}{b_{i,\tau }^{v}}+\underset{\nu }{\sum }\langle t_{\nu ,i}\rangle \right)}^{-1},\\ \text{exp}(\langle \text{log }v_{i,\tau }\rangle ) & =\text{exp}(\Psi (\alpha_{i,\tau }^{v}))\beta_{i,\tau }^{v},\\ \langle v_{i,\tau }\rangle & =\alpha_{i,\tau }^{v}\beta_{i,\tau }^{v}. \end{array}$$

### 3.3. Efficient Implementation

One of the attractive features of NMF is easy and efficient implementation. In this section, we derive that the update equations of Section 3.2 in compact matrix notation are to illustrate that these attractive properties are retained for the full Bayesian treatment. A subtle but key point in the efficiency of the algorithm is that we can avoid explicitly storing and computing the *W* × *I* × *K* object 〈*S*〉, as we only need the marginal statistics during optimisation. Consider (33). We can write

(35) $$\begin{array}{ll} \underset{\tau }{\sum }\langle s_{\nu ,i,\tau }\rangle & =\underset{\tau }{\sum }x_{\nu ,\tau }p_{\nu ,i,\tau }\\ & =\text{exp}(\langle \text{log }t_{\nu ,i}\rangle )\\ & \;\;\times \underset{\tau }{\sum }\left(\frac{x_{\nu ,\tau }}{\left(\sum_{i'}\text{exp}(\langle \text{log }t_{\nu ,i'}\rangle )\text{exp}(\langle \text{log }v_{i',\tau }\rangle )\right)}\right)\\ & \;\;\times \text{exp}(\langle \text{log }v_{i,\tau }\rangle ),\\ \Sigma_{t} & =L_{t}\;\;.*((X./(L_{t}L_{v}))L_{v}^{⊤}). \end{array}$$

Here, the denominator has to be nonzero. In the last line, we have represented the expression in compact notation where we define the following matrices:

(36) $$\begin{array}{c} E_{t}=\{\langle t_{\nu ,i}\rangle \},\;\;L_{t}=\{\text{exp}(\langle \text{log }t_{\nu ,i}\rangle )\},\\ \Sigma_{t}=\{\underset{\tau }{\sum }\langle s_{\nu ,i,\tau }\rangle \},\;\;A_{t}=\{a_{\nu ,i}^{t}\},\\ B_{t}=\{b_{\nu ,i}^{t}\},\;\;\alpha_{t}=\{\alpha_{\nu ,i}^{t}\},\;\;\beta_{t}=\{\beta_{\nu ,i}^{t}\},\\ E_{v}=\{\langle v_{i,\tau }\rangle \},\;\;L_{v}=\{\text{exp}(\langle \text{log}\;v_{i,\tau }\rangle )\},\\ \Sigma_{v}=\{\underset{\nu }{\sum }\langle s_{\nu ,i,\tau }\rangle \},\;\;A_{v}=\{a_{i,\tau }^{v}\},\\ B_{v}=\{b_{i,\tau }^{v}\},\;\;\alpha_{v}=\{\alpha_{i,\tau }^{v}\},\;\;\beta_{v}=\{\beta_{i,\tau }^{v}\}. \end{array}$$

The matrices subscripted with *t* are in ℝ_ + _^*W*×*I*^ and with *v* are in ℝ_ + _^*I*×*K*^. For notational convenience, we define. ∗ and ./ as elementwise matrix multiplication and division, respectively, and **1**_*W*_ as a *W* × 1 vector of ones. After straightforward substitutions, we obtain the *variational nonnegative matrix factorisation* algorithm, that can compactly be expressed as in panel Algorithm 1.

Similarly, an iterative conditional modes (ICM) algorithm can be derived to compute the maximum a posteriori (MAP) solution (see Appendix A.4):

(37) $$\begin{array}{ll} V & :=(A_{v}+V\;.*(T^{⊤}((M\;.*X)./(TV))))\\ & \;./(A_{v}./B_{v}+T^{⊤}M), \end{array}$$

(38) $$\begin{array}{ll} T & :=(A_{t}+T\;.*(((M\;.*X)./(TV))V^{⊤}))\\ & \;./(A_{t}./B_{t}+MV^{⊤}). \end{array}$$

Note that when the shape parameters go to zero, that is, *A*_*t*_, *A*_*v*_ → **0**, we obtain the maximum likelihood NMF algorithm.

### 3.4. Markov Chain Monte Carlo, the Gibbs Sampler

Monte Carlo methods [15, 16] are powerful computational techniques to estimate expectations of form

(39) $$E={\langle f\left(x\right)\rangle }_{p(x)}\approx \frac{1}{N}\overset{N}{\underset{n=1}{\sum }}f(x^{\left(i\right)})={\overset{˜}{E}}_{N},$$

where *x* ^(*i*)^ are independent samples drawn from *p*(*x*). Under mild conditions on *f*, the estimate ${\tilde{E}}_{N}$ converges to the true expectation for *N* → ∞. The difficulty here is obtaining independent samples {*x* ^(*i*)^}_*i*=1 ⋯ *N*_ from complicated distributions.

The Markov Chain Monte Carlo (MCMC) techniques generate subsequent samples from a Markov chain defined by a *transition kernel* *𝒯*, that is, one generates *x* ^(*i*+1)^ conditioned on *x* ^(*i*)^ as follows:

(40) $$x^{\left(i+1\right)}∼𝒯(x\;|\;x^{\left(i\right)}).$$

Note that the transition kernel *𝒯* is not needed explicitly in practice; all is needed is a procedure to sample a new configuration, given the previous one. Perhaps surprisingly, even though subsequent samples are correlated, provided that *𝒯* satisfies certain ergodicity conditions, (39) remains still valid, and estimated expectations converge to their true values when number of samples *N* goes to infinity [15]. To design a transition kernel *𝒯* such that the desired distribution is the stationary distribution, that is, *p*(*x*) = ∫ *d* *x*′*𝒯*(*x* ∣ *x*′)*p*(*x*′), many alternative strategies can be employed [16]. One particularly convenient and simple procedure is the Gibbs sampler where one samples each block of variables from *full conditional distributions*. For the NMF model, a possible Gibbs sampler is

(41) $$\begin{array}{c} S^{\left(n+1\right)}∼p(S\;|\;T^{\left(n\right)},V^{\left(n\right)},X,\Theta ),\\ T^{\left(n+1\right)}∼p(T\;|\;V^{\left(n\right)},S^{\left(n+1\right)},X,\Theta ),\\ V^{\left(n+1\right)}∼p(V\;|\;S^{\left(n+1\right)},T^{\left(n+1\right)},X,\Theta ). \end{array}$$

Note that this procedure implicitly defines a transition kernel *𝒯*(· ∣ ·). It can be shown [15] that the stationary distribution of *𝒯* is the exact posterior *p*(*S*, *T*, *V* ∣ *X*, Θ). Eventually, the Gibbs sampler converges regardless of the order that the blocks are visited, provided that each block is visited infinitely often in the limit *n* → ∞. However, the rate of convergence is very difficult to assess as it depends upon the order of the updates as well as the starting configuration (*T* ^(0)^, *V* ^(0)^, *S* ^(0)^). It is instructive to contrast above (41) with the variational update of (30)–(32): algorithmically the two approaches are quite similar. The pseudo-code is given in Algorithm 2.

#### 3.4.1. Marginal Likelihood Estimation with Chib's Method

The marginal likelihood can be estimated from the samples generated by the Gibbs sampler using a method proposed by Chib [17]. Suppose we have run the block Gibbs sampler until convergence and have *N* samples as follows:

(42) $${\left\{T^{\left(n\right)}\right\}}_{n=1\;\;:\;N},\;\;{\left\{V^{\left(n\right)}\right\}}_{n=1\;\;:\;N},\;\;{\left\{S^{\left(n\right)}\right\}}_{n=1\;\;:\;N}.$$

The marginal likelihood is (omitting hyperparameters Θ)

(43) $$p(X)=\frac{p(V,T,S,X)}{p(V,T,S\;|\;X)}.$$

This equation holds for *all* points (*V*, *T*, *S*). We choose a point in the configuration space; provided that the distribution is unimodal, a good candidate is a configuration near the mode $\left(\tilde{T},\tilde{V},\tilde{S}\right)$. The numerator in (43) is easy to evaluate. The denominator is

(44) $$\begin{array}{ll} p(\overset{˜}{V},\overset{˜}{T},\overset{˜}{S}\;|\;X) & =p(\overset{˜}{V}\;|\;\overset{˜}{T},\overset{˜}{S},X)p(\overset{˜}{T}\;|\;\overset{˜}{S},X)p(\overset{˜}{S}\;|\;X)\\ & =p(\overset{˜}{V}\;|\;\overset{˜}{T},\overset{˜}{S})p(\overset{˜}{T}\;|\;\overset{˜}{S})p(\overset{˜}{S}\;|\;X). \end{array}$$

The first term is full conditional, so it is available for the Gibbs sampler. The third term is

(45) $$\begin{array}{ll} p(\overset{˜}{S}\;|\;X) & =\int dV\;\;dT\;\;p(\overset{˜}{S}\;|\;V,T,X)p(V,T\;|\;X)\\ & \approx \frac{1}{N}\overset{N}{\underset{n=1}{\sum }}p(\overset{˜}{S}\;|\;V^{\left(n\right)},T^{\left(n\right)},X). \end{array}$$

The second term is trickier

(46) $$p(\overset{˜}{T}\;|\;\overset{˜}{S})=\int dV\;\;p(\overset{˜}{T}\;|\;V,\overset{˜}{S})p(V\;|\;\overset{˜}{S}).$$

The first term here is full conditional. However, the original Gibbs run gives us only samples from *p*(*V* ∣ *X*), not $p(V\;|\;\overset{˜}{S})$. The idea is to run the Gibbs sampler for *M* further iterations where we sample from $\left(V_{\overset{˜}{S}}^{\left(m\right)},T_{\overset{˜}{S}}^{\left(m\right)})∼p(V,T\;|\;S=\overset{˜}{S}\right)$, that is, with *S* clamped at $\tilde{S}$. The resulting estimate is

(47) $$p(\overset{˜}{T}\;|\;\overset{˜}{S})\approx \frac{1}{M}\overset{M}{\underset{m=1}{\sum }}p(\overset{˜}{T}\;|\;V_{\overset{˜}{S}}^{\left(m\right)},\overset{˜}{S}).$$

Chib's method estimates the marginal likelihood as follows:

(48) $$\begin{array}{ll} \text{log}\;p(X\;|\;\Theta ) & =\text{log}\;p(\overset{˜}{V},\overset{˜}{T},\overset{˜}{S},X\;|\;\Theta )-\text{log}\;p(\overset{˜}{V},\overset{˜}{T},\overset{˜}{S}\;|\;X,\Theta )\\ & \approx \text{log}\;p(\overset{˜}{V},\overset{˜}{T},\overset{˜}{S},X\;|\;\Theta )-\text{log}\;p(\overset{˜}{V}\;|\;\overset{˜}{T},\overset{˜}{S},\Theta )\\ & \;-\text{log}\;\overset{M}{\underset{m=1}{\sum }}p(\overset{˜}{T}\;|\;V_{\overset{˜}{S}}^{\left(m\right)},\overset{˜}{S},\Theta )\\ & \;-\log\overset{N}{\underset{n=1}{\sum }}p(\overset{˜}{S}\;|\;V^{\left(n\right)},T^{\left(n\right)},X,\Theta )+\text{log}(MN). \end{array}$$

## 4. Simulations

Our goal is to illustrate our approach in a model selection context. We first illustrate that the variational approximation to the marginal likelihood is close to the one obtained from the Gibbs sampler via Chib's method. Then, we compare the quality of solutions we obtain via Variational NMF and compare them to the original NMF on a prediction task. Finally, we focus on reconstruction quality in the overcomplete case where the standard NMF is subject to overfitting.

**Model Order Determination**

**Figure 3:**
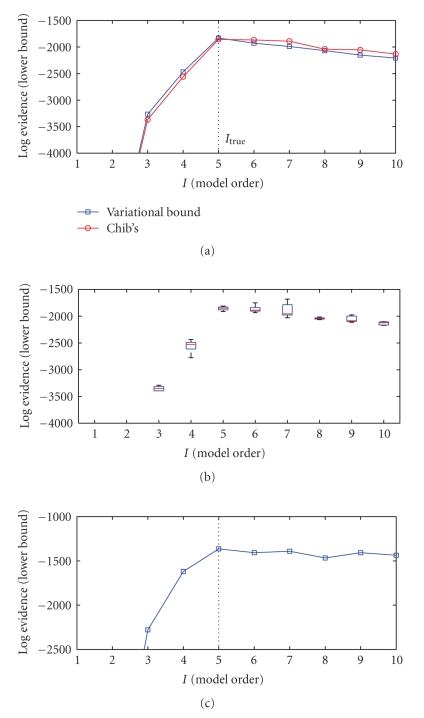
Model selection results. (a) Comparison of model selection by variational bound (squares) and marginal likelihood estimated by Chib's (circles) method. The hyperparameters are assumed to be known. (b) Box-plot of marginal likelihood estimated by Chib's method using 5000, 10 000, and 10 000 iterations for burn-in, free, and clamped sampling. The boxes show the lower quartile, median, and upper quartile values. (c) Model selection by variational bound when hyperparameters are unknown and jointly estimated.

**Figure 4:**
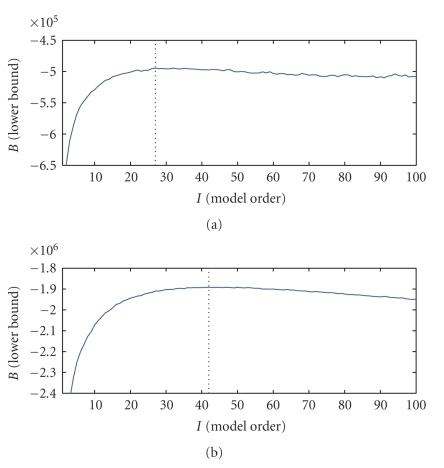
Model selection using variational bound with adapted hyperparameters on face data 16 × 16 with I* = 27 (a) and 32 × 32 with I* = 42 (b).

To test our approach, we generate synthetic data from the hierarchical model in (21) with *W* = 16, *K* = 10, and the number of sources *I*_true_ = 5. The inference task is to find the correct number of sources, given *X*. The hyperparameters of the true model are set to *a*_*ν*,*i*_^*t*^ = *a* ^*t*^ = 10, *b*_*ν*,*i*_^*t*^ = *b* ^*t*^ = 1, *a*_*i*,*τ*_^*v*^ = *a* ^*v*^ = 1, and *b*_*i*,*τ*_^*v*^ = *b* ^*v*^ = 100. In the first experiment, the hyperparameters are assumed to be known and in the second are jointly estimated from data, using hyperparameter adaptation. We evaluate the marginal likelihood for models with the number of templates *I* = 1 ⋯ 10, with the Gibbs sampler using Chib's method and variational lower bound *ℬ* via variational Bayes. We run the Gibbs sampler for MAXITER = 10 000 steps following a burn-in period of 5000 steps; then we clamp the sources *S* and continue the simulation for a further 10 000 steps to estimate quantities required by Chib's method. We run the variational algorithm until convergence of the bound or 10 000 iterations, whichever occurs first. In Figure 3(a), we show a comparison of the variational estimate with the average of 5 independent runs obtained via Chib's method. We observe, that both methods give consistent results. In Figure 4, we show the lower bound as a function of model order *I*, where for each *I*, the bound is optimised independently by jointly optimising hyperparameters *a*_*t*_, *b*_*t*_, *a*_*v*_, and *b*_*v*_ using the equations derived in the appendix. We observe, that the correct model order can be inferred even when the hyperparameters are unknown a priori. This is potentially useful for estimation of model order from real data.

As real data, we use a version of the Olivetti face image database (*K* = 400 images of 64 × 64 pixels available at http://www.cs.toronto.edu/*∼*roweis/data/olivettifaces.mat). We further downsampled to 16 × 16 or 32 × 32 pixels, hence our data matrix *X* is 16^2^ × 400 or 32^2^ × 400. We use a model with tied hyperparameters as *a*_*ν*,*i*_^*t*^ = *a* ^*t*^, *b*_*ν*,*i*_^*t*^ = *b* ^*t*^, *a*_*i*,*τ*_^*v*^ = *a* ^*v*^, and *b*_*i*,*τ*_^*v*^ = *b* ^*v*^, where all hyperparameters are jointly estimated. In Figure 4, bottom, we show results of model order determination for this dataset with joint hyperparameter adaptation. Here, we run the variational algorithm for each model order *I* = 1 ⋯ 100 independently and evaluate the lower bound after optimising the hyperparameters. The Gibbs sampler is not found practical and is omitted here. The lower bound behaves as is expected from marginal likelihood, reflecting the tradeoff between too many and too few templates. Higher resolution implies more templates, consistent with our intuition that detail requires more templates for accurate representation.

**Figure 5:**
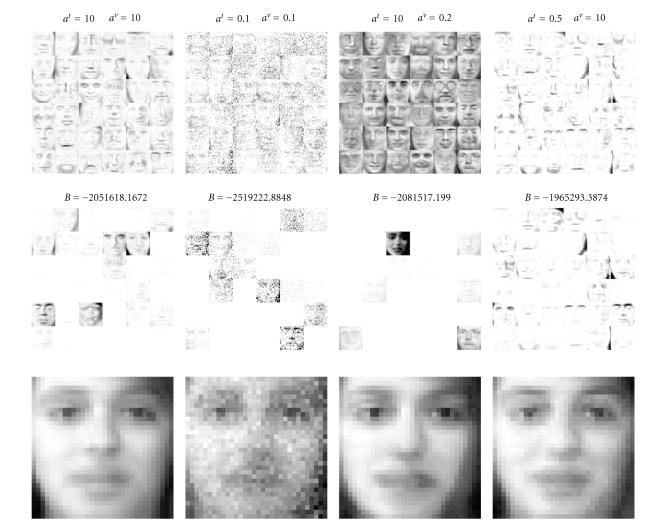
Templates, excitations for a particular example, and the reconstructions obtained for different hyperparameter settings. *B* is the lower bound for the whole dataset.

We also investigate the nature of the representations (see Figure 5). Here, for each independent run, we fix the values of shape parameters to (*a* ^*t*^, *a* ^*v*^) = [(10, 10), (0.1, 0.1), (10, 0.2), (10, 0.5)] and only estimate *b* ^*t*^ and *b* ^*v*^. This corresponds to enforcing sparse or nonsparse *t* and *v*. Each column shows *I* = 36 templates estimated from the dataset conditioned on hyperparameters. The middle image is the same template image above weighted with the excitations corresponding to the reconstruction (the expected value of the predictive distribution) below. Here, we clearly see the effect of the hyperparameters. In the first condition (*a* ^*t*^, *a* ^*v*^) = (10, 10), the prior does not enforces sparsity to the templates and excitations. Hence, for the representation of a given image, there are many active templates. In the second condition, we try to force both matrices to be sparse with (*a* ^*t*^, *a* ^*v*^) = (0.1, 0.1). Here, the result is not satisfactory as isolated components of the templates are zeroed, giving a representation that looks like one contaminated by “salt-and-pepper” noise. The third condition ((*a* ^*t*^, *a* ^*v*^) = (10, 0.2)) forces only the excitations to be sparse. Here, we observe that the templates correspond to some average face images. Qualitatively, each image is reconstructed using a superposition of a few of these templates. In the final representation, we enforce sparsity in the templates but not in the excitations. Here, our estimate finds templates that correspond to parts of individual face images (eyebrows, lips, etc.). This solution, intuitively corresponding to a parsimonious representation, also is the best in terms of the marginal likelihood. With proper initialisation, our variational procedure is able to find such solutions.

**Prediction**

We now compare variational Bayesian NMF with the maximum likelihood NMF on a missing data prediction task.

**Figure 6:**
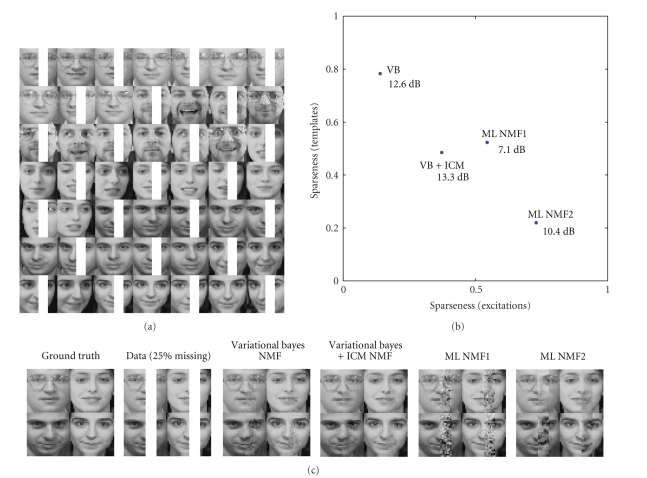
Results of a typical run. (a) Example images from the dataset. (b) Comparison of the reconstruction accuracy of different methods in terms of SNR (in dB), organised according to the sparseness of the solution. (c) (from left to right). The ground truth, data with missing pixels. The reconstructions of VB, VB + ICM, and ML-NMF with two initialisation strategies (1 = random, 2 = to image).

To illustrate the self regularisation effect, we set up an experiment in which we select a subset of the face data consisting of 50 images. From half of the images, we remove the same patch (Figure 6) and predict the missing pixels. This is a rather small dataset for this task, as we have only 10 images for each of the 5 different persons, and half of these images have missing data at the same spot. We measure the quality of the prediction in terms of signal-to-noise ratio (SNR). The missing values are reconstructed using the mean of the predictive distribution *X*_pred_ ≡ 〈*X*〉_*𝒫𝒪*(*X*;T*V*)_ = T*V*, where T* and V* are point estimates of the template and excitation matrix. We compare our variational algorithm with the classical NMF. For each algorithm, we test two different versions. The variational algorithms differ in how we estimate T* and V*. In the first variational algorithm, we use a crude estimate of T* and V* as the mean of the approximating *q* distribution. In the second condition, after convergence of hyperparameters via VB, we reinitialise *T* and *V* randomly and switch to an ICM algorithm (see (38)). This strategy finds a local mode (T*, V*) of the exact posterior distribution. In NMF, we test two initialisation strategies: in the first condition, we initialise the templates randomly; in the second, we set them equal to the images in the dataset with random perturbations.

In Figure 6, we show the reconstruction results for a typical run, for a model with *I* = 100 templates. Note that this is an overcomplete model, with twice as many templates as there are images. To characterise the nature of the estimated template and excitation matrices, we use the sparseness criteria [18] of an *m* × *n* matrix *X*, defined as $\text{Sparseness}\;(X)=(\sqrt{mn}-(\sum_{i,j}|X_{i,j}|)/(\sum_{i,j}X_{i,j}^{2}{)}^{1/2})/(\sqrt{mn}-1)$. This measure is 1 when the matrix *X* has only a single nonzero entry and 0 when all entries are equal. We see that the variational algorithms are superior in this case in terms of SNR as well as the visual quality of the reconstruction. This is perhaps not surprising, since with maximum likelihood estimation; if the model order is not carefully chosen, generalisation performance is poor: the “noise” in the observed data is fitted but the prediction quality drops on new data. An interesting observation is that highly sparse solutions (either in templates or excitations) do not give the best result; the solution that balances both seems to be the best in this setting. This example illustrates that sparseness in itself may not be necessarily a good criteria to optimise; for model selection, the marginal likelihood should be used as the natural quantity.

**Figure 7:**
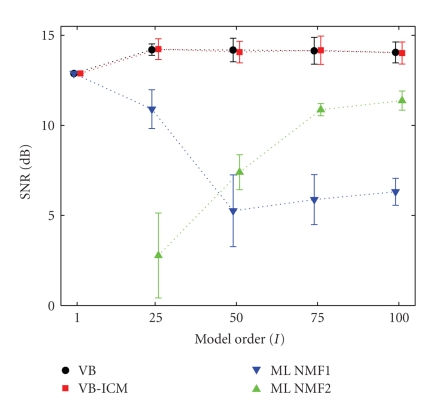
Average SNR results for model orders *I* = 1, 25, 50, 75, 100 covering undercomplete, complete, and overcomplete cases. Comparison of VB, VB + ICM, and ML-NMF with two initialisation strategies (1 = random, 2 = to image).

On the same face dataset, we compare the prediction error in terms of the SNR for varying model order *I*. Our goal is to compare the prediction performance of the full Bayesian approach with the ML-NMF for a range of conditions (under-complete, complete, and overcomplete). The results shown in Figure 7 are averages of several runs with hyperparameter adaptation and different hyperparameter tying. In the simulations, the shape parameters are tied always as *a*_*i*,*τ*_^*v*^ = *a* ^*v*^ (and *a*_*ν*,*i*_^*t*^ = *a* ^*t*^). The scale parameters are untied or tied as (*b*_*τ*_^*v*^, *b*_*ν*_^*t*^) (across sources) or *b*_*i*_^*v*^, *b*_*i*_^*t*^ (different for each source) and jointly optimised. Regardless of the hyperparameter tying structure, the results were quite similar. The best SNR values are attained with untied scale parameters for both excitations and templates.

We observe that, due to the implicit self-regularisation in the Bayesian approach, the prediction performance is not very sensitive to the model order and is immune to overfitting. In contrast, the ML-NMF with random initialisation is prone to overfitting, and prediction performance drops with increasing model order. Interestingly, when we initialise the ML-NMF algorithm to true data points with small perturbations, the prediction performance in terms of SNR improves. Note that this strategy would not be possible for data where the pixels were truly missing. However, visual inspection shows that the interpolation can still be “patchy” (see Figure 6).

We observe that hyperparameter adaptation is crucial for obtaining good prediction performance. In our simulations, results for VB without hyperparameter adaptation were occasionally poorer than the ML estimates. Good initialisation of the shape hyperparameters seems to be also important. We obtain best results when initialising the shape hyperparameters asymmetrically, for example, *a* ^*v*^ < 1 and *a* ^*t*^ > 10 (see 3rd and 4th panels from left in Figure 5). When the shape hyper-parameters are initialised to small *a* ^*v*^, *a* ^*t*^ ≪ 1, the EM seems to get stuck in a local minima more often. Consequently, the prediction results are poorer. We have also carried out tests with more undercomplete representations when the model order is low *I* < 10. For these simulations, while the marginal likelihood was in favour of the VB solutions, we have not observed statistically significant differences between VB and ML in terms of SNR. The SNR improvement of VB over ML was on average about 0.1 dB only.

## 5. Discussion and Conclusions

In this paper, we have investigated KL-NMF from a statistical perspective. We have shown that KL minimisation formulation the original algorithm can be derived from a probabilistic model where the observations are superposition of *I* independent Poisson-distributed latent sources. Here, the template and excitation matrices turn out to be latent intensity parameters. The interpretation of NMF as a maximum likelihood method in a Poisson model is mentioned in the original NMF paper [1] and discussed in more detail by [5, 10], and [5] focuses on the optimisation and gives an equivalent description using auxiliary function maximisation. In contrast, [10] illustrates that the auxiliary variables can be viewed as model variables (the sources *s*) that are analytically integrated out. The relationship between KL divergence and the Poisson distribution is not just a lucky coincidence. There exists a duality between divergence functions and exponential family distributions. If a cost function is a Bregman divergence, there exists a regular exponential family where minimising the cost corresponds to maximum likelihood parameter estimation [19]; also see [12] it in the context of matrix factorisation models.

The novel observation in the current article is the exact characterisation of the approximating distribution *q*(*S*) or full conditionals *p*(*S* ∣ *T*, *V*, *X*) as a product of multinomial distributions, leading to a richer approximation distribution than a naive mean field or single site Gibbs (which would freeze due to deterministic *p*(*X* ∣ *S*)). This conjugate form leads to significant simplifications in full Bayesian integration. Apart from the conditionally Gaussian case, NMF with KL objective seems to be unique in this respect. For several other distance metrics *D*(·||·), we find that full Bayesian inference not as practical as *p*(*S* ∣ *T*, *V*, *X*) is not standard.

We have also shown that the standard KL-NMF algorithm with multiplicative update rules is in fact an EM algorithm with data augmentation. Extending upon this observation, we have developed an hierarchical model with conjugate Gamma priors. We have developed a variational Bayes algorithm and a Gibbs sampler for inference in this hierarchical model. We have also developed methods for estimating the marginal likelihood for model selection. This is an additional feature that is lacking in existing NMF approaches with regularisation, where only MAP estimates are obtained, such as [13, 18, 20].

Our simulations suggest that the variational bound seems to be a reasonable approximation to the marginal likelihood and can guide model selection for NMF. The computational requirements are comparable to the ML-NMF. A potentially time-consuming step in the implementation of the variational algorithm is the evaluation of the Ψ function but this step can also be replaced by a simple piecewise polynomial approximation since exp(Ψ(*x*)) ≈ *x* − 0.5 for *x* > 5.

We first compare the variational inference with a Gibbs sampler. In our simulations, we observe that both algorithms give qualitatively very similar results, both for inference of templates and excitations as well as model order selection. We find the variational approach somewhat more practical as it can be expressed as simple matrix operations, where both the fixed point equations as well as the bound can be compactly and efficiently implemented using matrix computation software. In contrast, our Gibbs sampler is computationally more demanding, and the calculation of marginal likelihood is somewhat more tricky. With our implementation of both algorithms, the variational method is faster by a factor of around 13. Reference implementations of both algorithms in Matlab are available from the following url: http://www.cmpe.boun.edu.tr/*∼*cemgil/bnmf/.

In terms of computational requirements, the variational procedure has several advantages. First, we circumvent sampling from multinomial variables, which is the main computational bottleneck with the Gibbs sampler. Whilst efficient algorithms are developed for multinomial sampling [21], the procedure is time consuming when the number of latent sources *I* is large. In contrast, the variational method estimates the expected sufficient statistics directly by elementary matrix operations. Another advantage is hyperparameter estimation. In principle, it is possible to maximise the marginal likelihood via a Monte Carlo EM procedure [22, 23]; yet this potentially requires many more iterations. In contrast, the evaluation of the derivatives of the lower bound is straightforward and can be implemented without much additional computational cost.

The efficiency of the Gibbs sampler could be improved by working out the distribution of the sufficient statistics of sources directly (namely, quantities ∑_*τ*_ *s*_*ν*,*i*,*τ*_ or ∑_*ν*_ *s*_*ν*,*i*,*τ*_) to circumvent multinomial sampling. Unfortunately, for the sum of binomial random variables with different cell probability parameters, the sum does not have a simple form but various approximations are possible [24].

Inference based on VB is easy to implement but at the end of the day, the fixed point iteration is just a gradient-based lower bound optimisation procedure, and second order Newton methods can provide more efficient alternatives. For NMF models, there exist many conditional independence relations, hence the Hessian matrix has a special block structure [12]. It is certainly interesting to develop efficient inference methods that make use of the special block structure of the Hessian matrix. However, as our primary goal was a practical full Bayesian treatment, we have not investigated this path yet. Another approach in this direction is using alternative deterministic integration techniques such as expectation propagation (EP) [25]. Those techniques work directly on an approximation of the true marginal likelihood rather than a bound. A related approach known as expectation consistent (EC) inference is used with success in related source separation problems [26].

From a modelling perspective, our hierarchical model provides some attractive properties. It is easy to incorporate prior knowledge about individual latent sources via hyperparameters, and one can easily capture variability in the templates and excitations that is potentially useful for developing robust techniques. The prior structure here is qualitatively similar to an entropic prior [20, 27], and we find qualitatively similar representations to the ones found by NMF reported earlier by [1, 18]. However, none of the above mentioned methods provide an estimate of the marginal likelihood, which is useful for model selection. Our generative model formulation can be extended in various ways to suit the specific needs of particular applications. For example, one can enforce more structured prior models such as chains or fields [10]. As a second possibility, the Poisson observation model can be replaced with other models such as clipped Gaussian, Gamma, or Gaussians which lead to alternative source separation algorithms. For example, the case of Gaussian sources where the excitations and templates correspond to the variances is discussed in [28].

Our main contribution here is the development of a principled and practical way to estimate both the optimal sparsity criteria and model order, in terms of marginal likelihood. By maximising the bound on marginal likelihood, we have a method where all the hyperparameters can be estimated from data, and the appropriate sparseness criteria is found automatically. We believe that our approach provides a practical improvement to the highly popular KL-NMF algorithm without incurring much additional computational cost.

## Figures and Tables

**Algorithm 1:**
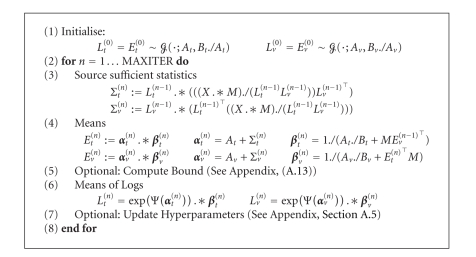
Variational nonnegative matrix factorisation.

**Algorithm 2:**
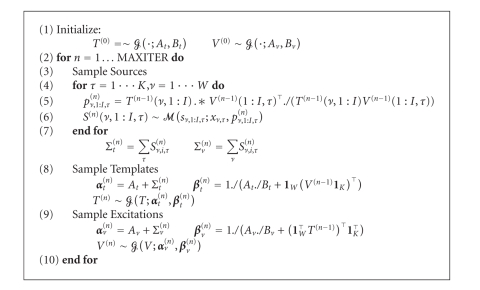
Gibbs sampler for nonnegative matrix factorisation.

## Appendix

### A. Standard Distributions in Exponential Form, Their Sufficient Statistics and Entropies

- Gamma
  (A.1) $$\begin{array}{c} 𝒢(\lambda ;a,b)\equiv \text{exp}\left(+(a-1)\log\lambda -\frac{1}{b}\lambda -\log\Gamma (a)-a\log b\right),\\ {\langle \lambda \rangle }_{𝒢}=ab\;\;{\langle \text{log}\;\lambda \rangle }_{𝒢}=\Psi (a)+\text{log}(b),\\ H_{𝒢}[\lambda ]\equiv -{\langle \text{log}\;𝒢\rangle }_{𝒢}=-(a-1)\Psi (a)+\text{log}\;b+a+\text{log}\;\Gamma (a). \end{array}$$
  Here, Ψ denotes the digamma function defined as Ψ(*a*) ≡ *d*logΓ(*a*)/*da*.
- Poisson
  (A.2) $$\begin{array}{ll} 𝒫𝒪(s;\lambda ) & =\text{exp}(s\;\;\text{log}\;\;\lambda -\lambda -\text{log}\;\Gamma (s+1)),\\ {\langle s\rangle }_{𝒫𝒪} & =\lambda . \end{array}$$
- Multinomial
  (A.3) $$ℳ(s;x,p)=\delta (x-\underset{i}{\sum }s_{i})\text{exp}\left(\text{log}\;\Gamma (x+1)+\overset{I}{\underset{i=1}{\sum }}\left(s_{i}\;\;\text{log}\;p_{i}-\text{log}\;\Gamma (s_{i}+1)\right)\right),{\langle s_{i}\rangle }_{ℳ}=xp_{i}.$$
  Here, **s** = {*s*_1_, *s*_2_,…, *s*_*I*_}, **p** = {*p*_1_, *p*_2_,…, *p*_*I*_}, and *p*_1_ + *p*_2_ + ⋯ + *p*_*I*_ = 1. Here, *p*_*i*_, *i* = 1 ⋯ *I* are the cell probabilities, and *x* is the index parameter where *s*_1_ + *s*_2_ + ⋯ + *s*_*I*_ = *x*. The entropy is given as follows:
  (A.4) $$\begin{array}{ll} H_{ℳ}[s_{\nu ,1:I,\tau }] & =-\text{log}\;\Gamma (x_{\nu ,\tau }+1)-\overset{I}{\underset{i=1}{\sum }}\langle s_{\nu ,i,\tau }\rangle \text{log}\;p_{\nu ,i,\tau }\\ & \;+\overset{I}{\underset{i=1}{\sum }}\langle \text{log }\Gamma (s_{\nu ,i,\tau }+1)\rangle -\langle \text{log}\;\delta (x_{\nu ,\tau }-\underset{i}{\sum }s_{\nu ,i,\tau })\rangle . \end{array}$$

A closed form expression for the entropy is not known due to 〈logΓ(*s* + 1)〉 terms but asymptotic expansions exist [29, 30]. Computationally efficient sampling from a multinomial distribution is not trivial; see [21] for a comparison of various methods and detailed discussion of tradeoffs.

#### A.1. Summary of the Generative Model

We have the following. Indices:

*i* = 1 ⋯ *I*, source index;
*ν* = 1 ⋯ *W*, Row (frequency bin) index;
*τ* = 1 ⋯ *K*, Column (time frame) index;

*t*_*ν*,*i*_: template variable at *ν*th row of the *i*th source

(A.5) $$t_{\nu ,i}∼𝒢(t_{\nu ,i};a_{\nu ,i}^{t},\frac{b_{\nu ,i}^{t}}{a_{\nu ,i}^{t}});$$

*v*_*i*,*τ*_: excitation variable of the *i*th source at *τ*th column

(A.6) $$v_{i,\tau }∼𝒢(v_{i,\tau };a_{i,\tau }^{v},\frac{b_{i,\tau }^{v}}{a_{i,\tau }^{v}});$$

*s*_*ν*,*i*,*τ*_: source variable of *i*th source at *ν*th row (frequency bin) and *τ*th column (time frame)

(A.7) $$s_{\nu ,i,\tau }∼𝒫𝒪(s_{\nu ,i,\tau };t_{\nu ,i}v_{i,\tau });$$

*x*_*ν*,*τ*_: observation at *ν*th row (frequency bin) and *τ*th column (time frame)

(A.8) $$x_{\nu ,\tau }∼\underset{i}{\sum }s_{\nu ,i,\tau }.$$

#### A.2. Expression of the Full Joint Distribution

Here, *ϕ* ≡ *p*(*X*, *S*, *T*, *V* ∣ Θ) = *p*(*X* ∣ *S*)*p*(*S* ∣ *T*, *V*)*p*(*V* ∣ Θ^*v*^)*p*(*V* ∣ Θ^*t*^),

(A.9) $$\begin{array}{ll} \text{log}\;\;\phi & =\underset{\nu }{\sum }\;\underset{i}{\sum }\;\underset{\tau }{\sum }\left(-t_{\nu ,i}v_{i,\tau }+s_{\nu ,i,\tau }\;\;\text{log}(t_{\nu ,i}v_{i,\tau })-\text{log}\;\Gamma (s_{\nu ,i,\tau }+1)\right)\\ & \;+\underset{\nu }{\sum }\underset{\tau }{\sum }\text{log}\;\delta (x_{\nu ,\tau }-\underset{i}{\sum }s_{\nu ,i,\tau })\\ & \;+\underset{\nu }{\sum }\;\underset{i}{\sum }+(a_{\nu ,i}^{t}-1)\text{log}\;t_{\nu ,i}-\frac{a_{\nu ,i}^{t}}{b_{\nu ,i}^{t}}t_{\nu ,i}-\text{log}\;\Gamma (a_{\nu ,i}^{t})\\ & \;-a_{\nu ,i}^{t}\;\;\text{log}\;(\frac{b_{\nu ,i}^{t}}{a_{\nu ,i}^{t}})\\ & \;+\underset{\tau }{\sum }\;\underset{i}{\sum }+(a_{i,\tau }^{v}-1)\text{log}\;v_{i,\tau }-\frac{a_{i,\tau }^{v}}{b_{i,\tau }^{v}}v_{i,\tau }-\text{log}\;\Gamma (a_{i,\tau }^{v})\\ & \;-a_{i,\tau }^{v}\;\;\text{log}(\frac{b_{i,\tau }^{v}}{a_{i,\tau }^{v}}). \end{array}$$

#### A.3. The Variational Bound

The variational bound in (27) can be written as

(A.10) $${ℒ}_{X}(\Theta )\equiv \text{log}\;p(X\;|\;\Theta )\geq {\langle \text{log}\;\phi \rangle }_{q}+H[q]={ℬ}_{\text{VB}},$$

where the energy term is given by the expectation of the expression in Appendix A.2, and *H*[*q*] denotes the entropy of the variational approximation distribution *q* where the individual entropies are defined in Appendix A:

(A.11) $$\begin{array}{ll} H[q] & =-\langle \text{log}\;q\rangle \\ & =\underset{\nu }{\sum }\;\underset{\tau }{\sum }H_{ℳ}[s_{\nu ,1:I,\tau }]+\underset{\nu }{\sum }\;\underset{i}{\sum }H_{𝒢}[t_{\nu ,i}]+\underset{i}{\sum }\;\underset{\tau }{\sum }H_{𝒢}[v_{i,\tau }]. \end{array}$$

One potential problem is that this expression requires the entropy of a multinomial distribution for which there is no known simple expression. This is due to terms of form 〈logΓ(*s* + 1)〉 where only asymptotic expansions are known. Fortunately, the difficult terms in the energy term can be canceled by the corresponding terms in the entropy term, and one obtains the following expression that only depends on known sufficient statistics:

(A.12) $$\begin{array}{ll} ℬ & =-\underset{\nu }{\sum }\;\underset{\tau }{\sum }\;\underset{i}{\sum }\langle t_{\nu ,i}\rangle \langle v_{i,\tau }\rangle \\ & \;+\underset{\nu }{\sum }\;\underset{i}{\sum }\langle \text{log}\;t_{\nu ,i}\rangle \left(a_{\nu ,i}^{t}-1+\underset{\tau }{\sum }\langle s_{\nu ,i,\tau }\rangle \right)\\ & \;+\underset{\tau }{\sum }\;\underset{i}{\sum }\langle \text{log}\;v_{i,\tau }\rangle \left(a_{i,\tau }^{v}-1+\underset{\nu }{\sum }\langle s_{\nu ,i,\tau }\rangle \right)\\ & \;+\underset{\nu }{\sum }\;\underset{i}{\sum }-\frac{a_{\nu ,i}^{t}}{b_{\nu ,i}}\langle t_{\nu ,i}\rangle -\text{log}\;\Gamma (a_{\nu ,i}^{t})-a_{\nu ,i}^{t}\;\;\;\text{log}\;(\frac{b_{\nu ,i}}{a_{\nu ,i}^{t}})\\ & \;+\underset{\tau }{\sum }\;\underset{i}{\sum }-\frac{a_{i,\tau }^{v}}{b_{i,\tau }^{v}}\langle v_{i,\tau }\rangle -\text{log}\;\Gamma (a_{i,\tau }^{v})-a_{i,\tau }^{v}\;\;\text{log}(\frac{b_{i,\tau }^{v}}{a_{i,\tau }^{v}})\\ & \;+\underset{\nu }{\sum }\;\underset{\tau }{\sum }\left(-\text{log}\;\Gamma (x_{\nu ,\tau }+1)-\overset{I}{\underset{i=1}{\sum }}\langle s_{\nu ,i,\tau }\rangle \text{log}\;p_{\nu ,i,\tau }\right)\\ & \;+\underset{\nu }{\sum }\;\underset{i}{\sum }\left(-(\alpha_{\nu ,i}^{t}-1)\Psi (\alpha_{\nu ,i}^{t})+\text{log}\;\beta_{\nu ,i}^{t}+\alpha_{\nu ,i}^{t}+\text{log}\;\Gamma (\alpha_{\nu ,i}^{t})\right)\\ & \;+\underset{i}{\sum }\;\underset{\tau }{\sum }\left(-(\alpha_{i,\tau }^{v}-1)\Psi (\alpha_{i,\tau }^{v})+\text{log}\;\beta_{i,\tau }^{v}+\alpha_{i,\tau }^{v}+\text{log}\;\Gamma (\alpha_{i,\tau }^{v})\right). \end{array}$$

After some careful manipulations, the following expression is obtained where log *L* denotes here elementwise logarithm of matrix *L*:

(A.13) $$\begin{array}{ll} ℬ & =\underset{\nu }{\sum }\;\underset{\tau }{\sum }\left(-E_{t}E_{v}-\text{log}\;\Gamma (X+1)\right)\\ & \;+\underset{\nu }{\sum }\;\underset{\tau }{\sum }-X\;.*(\left((L_{t}\;\;.*\;\text{log}(L_{t}))L_{v}+L_{t}(L_{v}\;.*\;\;\text{log}(L_{v}))\right)\\ & \;\;\;\;\;\;\;./(L_{t}L_{v})-\text{log}(L_{t}L_{v}))\\ & \;+\underset{\nu }{\sum }\;\underset{i}{\sum }-(A_{t}./B_{t})\;.*E_{t}-\text{log}\;\Gamma (A_{t})+A_{t}\;.*\text{log}(A_{t}./B_{t})\\ & \;+\underset{\nu }{\sum }\;\underset{i}{\sum }\alpha_{t}\;.*(\text{log}\;\beta_{t}+1)+\text{log}\;\Gamma (\alpha_{t})\\ & \;+\underset{i}{\sum }\;\underset{\tau }{\sum }-(A_{v}./B_{v})\;.*E_{v}-\text{log}\;\Gamma (A_{v})+A_{v}\;\;.*\text{log}(A_{v}./B_{v})\\ & \;+\underset{i}{\sum }\;\underset{\tau }{\sum }\alpha_{v}\;.*(\log\;\;\beta_{v}+1)+\log\;\;\Gamma (\alpha_{v}). \end{array}$$

#### A.4. Handling Missing Data and Map Estimation

When there is missing data, that is, when some of the *x*_*ν*,*τ*_ are not observed, computation is still straightforward in our framework and can be accomplished by a simple modification to the original algorithm. We first define a *mask* matrix **M** = {*m*_*ν*,*τ*_}, same size as *X*, where

(A.14) $$m_{\nu ,\tau }=\{\begin{array}{cc} 0, & x_{\nu ,\tau }\;\;\;\text{is}\;\;\text{missing},\\ 1, & \text{otherwise}. \end{array}$$

Using the mask variables, the observation model with missing data can be written as follows:

(A.15) $$p(X\;|\;S)p(S\;|\;T,V)=\underset{\nu ,\tau }{\prod }{\left(p(x_{\nu ,\tau }\;|\;s_{\nu ,1:I,\tau })p(s_{\nu ,1:I,\tau }\;|\;t_{\nu ,1:I},v_{1:I,\tau })\right)}^{m_{\nu ,\tau }}.$$

The prior is not affected. Hence, we merely replace the first two lines of the expression for the full joint distribution (given in the Appendix A.2) as follows:

(A.16) $$\begin{array}{ll} \text{log}\;\phi & =\underset{\nu }{\sum }\underset{\tau }{\sum }m_{\nu ,\tau }\underset{i}{\sum }\left(-t_{\nu ,i}v_{i,\tau }+s_{\nu ,i,\tau }\;\;\text{log}(t_{\nu ,i}v_{i,\tau }\right)\\ & \;\;\;\;\;\;\;-\text{log}\;\Gamma (s_{\nu ,i,\tau }+1))\\ & \;+\underset{\nu }{\sum }\underset{\tau }{\sum }m_{\nu ,\tau }\;\;\text{log}\;\delta (x_{\nu ,\tau }-\underset{i}{\sum }s_{\nu ,i,\tau })+\cdots . \end{array}$$

Consequently, it is easy to see that

(A.17) $$\begin{array}{cc} q(v_{i,\tau }) & \propto \text{exp}((a_{i,\tau }^{v}+\underset{\nu }{\sum }m_{\nu ,\tau }\langle s_{\nu ,i,\tau }\rangle -1)\log v_{i,\tau }\\ & \;\;-\left(\frac{a_{i,\tau }^{v}}{b_{i,\tau }^{v}}+\underset{\nu }{\sum }m_{\nu ,\tau }\langle t_{\nu ,i}\rangle \right)v_{i,\tau })\\ & \propto 𝒢\left(v_{i,\tau };\alpha_{i,\tau }^{v},\beta_{i,\tau }^{v}\right),\\ \alpha_{i,\tau }^{v} & \equiv a_{i,\tau }^{v}+\underset{\nu }{\sum }m_{\nu ,\tau }\langle s_{\nu ,i,\tau }\rangle ,\\ \beta_{i,\tau }^{v} & \equiv {\left(\frac{a_{i,\tau }^{v}}{b_{i,\tau }^{v}}+\underset{\nu }{\sum }m_{\nu ,\tau }\langle t_{\nu ,i}\rangle \right)}^{-1}. \end{array}$$

By a derivation analogous to one detailed in Section 3.3, we see that the excitation update equations in Algorithm 1, line 4, can be written using matrix notation as follows:

(A.18) $$\begin{array}{c} \Sigma_{v}=L_{v}\;.*(L_{t}^{⊤}((M\;.*X)./(L_{t}L_{v})))\\ \alpha_{v}=A_{v}+\Sigma_{v},\;\;\beta_{v}=1./\left(A_{v}./B_{v}+E_{t}^{⊤}M\right). \end{array}$$

The update rules for the templates are similar. Note that when there is no missing data, we have **M** = **1**_*W*_ **1**_*K*_^⊤^ which gives the original algorithm. The bound in (A.13) can also be easily modified for handling missing data. We merely replace **X** ← **M**. ∗**X** and the first term *E*_*t*_ *E*_*v*_ ← **M**. ∗*E*_*t*_ *E*_*v*_.

We conclude this subsection by noting that the standard NMF update equations, given in (20), can be also rewritten to handle missing data as follows:

(A.19) $$\begin{array}{l} V^{\left(n+1\right)}=V^{\left(n\right)}\;.*(T^{⊤}((M\;.*X)./(TV)))./(T^{⊤}M),\\ T^{\left(n+1\right)}=T^{\left(n\right)}\;.*(((M\;.*X)./(TV))V^{⊤})./(MV^{⊤}). \end{array}$$

Here, the denominator has to be nonzero. Similarly, an iterative conditional modes (ICM) algorithm can be derived to compute the maximum a posteriori (MAP) solution as follows:

(A.20) $$\begin{array}{ll} V^{\left(n+1\right)} & =(A_{v}+V^{\left(n\right)}\;.*(T^{⊤}((M\;.*X)./(TV))))\\ & \;./(A_{v}./B_{v}+T^{⊤}M),\\ T^{\left(n+1\right)} & =(A_{t}+T^{\left(n\right)}\;.*(((M\;.*X)./(TV))V^{⊤}))\\ & \;./(A_{t}./B_{t}+MV^{⊤}). \end{array}$$

Note that when the shape parameters go to zero, that is, *A*_*t*_, *A*_*v*_ → **0**, we obtain the maximum likelihood NMF algorithm.

#### A.5. Hyperparameter Optimisation

The hyperparameters Θ = (Θ^*t*^, Θ^*v*^) can be estimated by maximising the bound in 13. Below, we will derive the results for the excitations; the results for templates are similar. The solution for shape parameters involves finding the zero of a function *f*(*a*) − *c*, where

(A.21) $$\begin{array}{c} f(a)=\text{log}\;a-\Psi (a)+1,\\ a^{*}=f^{-1}(c). \end{array}$$

The solution can be found by Newton's method by iteration of the following fixed point equation:

(A.22) $$\begin{array}{ll} a^{\left(n+1\right)} & =a^{\left(n\right)}-\frac{f(a^{\left(n\right)})-c}{f^{\prime }(a^{\left(n\right)})}\\ & =a^{\left(n\right)}-\frac{\text{log}(a^{\left(n\right)})-\Psi (a^{\left(n\right)})+1-c}{1/a^{\left(n\right)}-\Psi^{\prime }(a^{\left(n\right)})}=a^{\left(n\right)}-\Delta^{\left(n\right)}. \end{array}$$

It is well known that Newton iterations can diverge if started away from the root. Occasionally, we observe that *a* can become negative. If this is the case, we set Δ^(*n*)^ ← Δ^(*n*)^/2, and try again. The digamma Ψ function and its derivative Ψ′ are available in numeric computation libraries (e.g., in Matlab as psi(0,a) and psi(1,a), resp.).

The derivation of the hyperparameter update equations is straightforward:

(A.23) $$\begin{array}{cc} \frac{\partial ℬ}{\partial a_{i,\tau }^{v}} & =\langle \text{log}\;v_{i,\tau }\rangle -\frac{1}{b_{i,\tau }^{v}}\langle v_{i,\tau }\rangle -\Psi (a_{i,\tau }^{v})\\ & \;-\text{log}\;b_{i,\tau }^{v}+\text{log}\;a_{i,\tau }^{v}+1=0,\\ c_{i,\tau } & =\text{log}\;a_{i,\tau }^{v}-\Psi (a_{i,\tau }^{v})+1,\\ c_{i,\tau } & \equiv \frac{\langle v_{i,\tau }\rangle }{b_{i,\tau }^{v}}-(\langle \text{log}\;v_{i,\tau }\rangle -\text{log}\;b_{i,\tau }^{v}),\\ \frac{\partial ℬ}{\partial b_{i,\tau }^{v}} & =\frac{a_{i,\tau }^{v}}{{\left(b_{i,\tau }^{v}\right)}^{2}}\langle v_{i,\tau }\rangle -a_{i,\tau }^{v}\frac{1}{b_{i,\tau }^{v}}=0,\\ b_{i,\tau }^{v} & =\langle v_{i,\tau }\rangle . \end{array}$$

Tying parameters across *τ* as *a*_*i*_^*v*^ = *a*_*i*,*τ*_^*v*^ and *b*_*i*_^*v*^ = *b*_*i*,*τ*_^*v*^ yields

(A.24) $$\begin{array}{cc} \frac{\partial ℬ}{\partial a_{i}^{v}} & =\underset{\tau }{\sum }\langle \log v_{i,\tau }\rangle -\underset{\tau }{\sum }\frac{1}{b_{i,\tau }^{v}}\langle v_{i,\tau }\rangle -K\Psi (a_{i}^{v})\\ & \;-\underset{\tau }{\sum }\text{log}\;b_{i,\tau }^{v}+K=0,\\ c_{i} & =\text{log}\;a_{i}^{v}-\Psi (a_{i}^{v})+1,\\ c_{i} & =\frac{1}{K}\underset{\tau }{\sum }\left(\frac{\langle v_{i,\tau }\rangle }{b_{i,\tau }^{v}}-\left(\langle \text{log }v_{i,\tau }\rangle -\text{log}\;b_{i,\tau }^{v}\right)\right),\\ \frac{\partial ℬ}{\partial b_{i}^{v}} & =\underset{\tau }{\sum }\frac{a_{i,\tau }^{v}}{{\left(b_{i}^{v}\right)}^{2}}\langle v_{i,\tau }\rangle -\frac{1}{b_{i}^{v}}\underset{\tau }{\sum }a_{i,\tau }^{v},\\ b_{i}^{v} & =\frac{\sum_{\tau }\;a_{i,\tau }^{v}\langle v_{i,\tau }\rangle }{\sum_{\tau }\;a_{i,\tau }^{v}}. \end{array}$$

Tying parameters across *τ* and *i*, *a* ^*v*^ = *a*_*i*,*τ*_^*v*^, and *b* ^*v*^ = *b*_*i*,*τ*_^*v*^ yields

(A.25) $$\begin{array}{c} c=\text{log}\;a^{v}-\Psi (a^{v})+1,\\ c=\frac{1}{KI}\underset{\tau }{\sum }\;\underset{i}{\sum }\left(\frac{\langle v_{i,\tau }\rangle }{b_{i,\tau }^{v}}-(\langle \text{log }v_{i,\tau }\rangle -\text{log}\;b_{i,\tau }^{v})\right),\\ \frac{\partial ℬ}{\partial b^{v}}=\underset{i}{\sum }\;\underset{\tau }{\sum }\frac{a_{i,\tau }^{v}}{{\left(b^{v}\right)}^{2}}\langle v_{i,\tau }\rangle -\frac{1}{b_{i}^{v}}\underset{i}{\sum }\;\underset{\tau }{\sum }a_{i,\tau }^{v},\\ b^{v}=\frac{\sum_{i}\;\sum_{\tau }\;a_{i,\tau }^{v}\langle v_{i,\tau }\rangle }{\sum_{i}\;\sum_{\tau }\;a_{i,\tau }^{v}}. \end{array}$$

The derivation of the template parameters is exactly analogous. We can express the update equations once again in compact matrix notation as follows:

(A.26) $$\begin{array}{ll} Z & ⟵E_{v}^{\left(n\right)}./B_{v}^{\left(n\right)}-\text{log}(L_{v}^{\left(n\right)}./B_{v}^{\left(n\right)}),\\ C & ⟵\left\{ \begin{array}{cc} Z, & \text{Not}\;\;\text{tied},\\ \frac{\left(Z1_{K}\right)}{K}, & \text{Tie}\;\;\;\text{columns}\;\;\;(\text{over}\;\;\;\;\tau ),\\ \frac{\left(1_{I}^{⊤}Z\right)}{I}, & \text{Tie}\;\;\;\text{rows}\;\;\;(\text{over}\;\;\;\;i),\\ \frac{\left(1_{I}^{⊤}Z1_{K}\right)}{\left(KI\right)}, & \text{Tie}\;\;\text{all}\;\;(\text{over}\;\;\;\tau \;\;\text{and}\;\;\;i), \end{array}\right.\\ A_{v}^{\left(n+1\right)} & ⟵\mathrm{SolveByNewton}\;\;(A_{v}^{\left(n\right)},C),\\ B_{v}^{\left(n+1\right)} & ⟵\left\{ \begin{array}{cc} E_{v}^{\left(n\right)}, & \text{Not}\;\;\text{tied},\\ \left(((A_{v}^{\left(n\right)}\;\;.*E_{v}^{\left(n\right)})1_{K}\right) & \\ \;\;./(A_{v}^{\left(n\right)}1_{K}))1_{K}^{⊤}, & \text{Tie}\;\;\;\text{columns}\;\;\;(\text{over}\;\;\;\;\tau ),\\ 1_{I}((1_{I}^{⊤}(A_{v}^{\left(n\right)}\;.*E_{v}^{\left(n\right)})) & \\ \;./(1_{I}^{⊤}A_{v}^{\left(n\right)})), & \text{Tie}\;\;\;\text{rows}\;\;\;(\text{over}\;\;\;\;i),\\ 1_{I}((1_{I}^{⊤}(A_{v}^{\left(n\right)}.*E_{v}^{\left(n\right)})1_{K}) & \\ \;\;./(1_{I}^{⊤}A_{v}^{\left(n\right)}1_{K}))1_{K}^{⊤}, & \text{Tie}\;\;\text{all}\;\;(\text{over}\;\;\;\tau \;\;\text{and}\;\;\;i). \end{array}\right. \end{array}$$

Here, we assume $\mathrm{SolveByNewton}(A_{0},C)$ is a matrix-valued function that finds root *C*_*i*,*j*_ = *f*(*A*_*i*,*j*_) for each element of *A*, starting from the initial matrix *A*_0_. If *C* is a scalar or vector, it is repeated over the missing index to implement parameter tying. For example, if *C* is a *I* × 1 vector and *A*_0_ is *I* × *K*, we assume *C*_*i*_ = *c*_*i*,*τ*_ for all *τ* = 1 ⋯ *K*, and the output is the same size as *A*_0_. This is only a notational convenience; an actual implementation can be achieved more efficiently. Again, the implementation of the template parameters is exactly analogous; merely replace above the subscripts as *v* ← *t*, (*i*, *τ*) ← (*ν*, *i*) and (*I*, *K*) ← (*W*, *I*).
